# Genetic Advances in Autism

**DOI:** 10.1007/s10803-020-04685-z

**Published:** 2020-09-17

**Authors:** Anita Thapar, Michael Rutter

**Affiliations:** 1 Division of Psychological Medicine and Clinical Neurosciences and MRC Centre for Neuropsychiatric Genetics and Genomics, Cardiff University, Hadyn Ellis Building, Cardiff, Maindy Road, Wales, CF24 4HQ UK; 2 Social, Genetic and Developmental Psychiatry Centre, Kings College London, London, UK

## Abstract

In the last 40 years, there has been a huge increase in autism genetics research and a rapidly growing number of discoveries. We now know autism is one of the most highly heritable disorders with negligible shared environmental contributions. Recent discoveries also show that rare variants of large effect size as well as small effect common gene variants all contribute to autism risk. These discoveries challenge traditional diagnostic boundaries and highlight huge heterogeneity in autism. In this review, we consider some of the key findings that are shaping current understanding of autism and what these discoveries mean for clinicians.

Over the last 40 years, our understanding of autism has evolved enormously. We have moved from a time when the role of genetics was unknown to an era when the first twin and family studies showed autism to be one of the most highly heritable disorders (Rutter 2011). These family-based studies motivated molecular genetic investigations, that most recently have led to an increasing number of reported autism gene discoveries and that are accompanied by a growing literature on potential biological insights. For those interested in details of autism risk loci, implicated genes and hypothesised biological mechanisms, the reader is directed to existing, comprehensive reviews on these topics (Vorstman et al. 2017; Sestan and State 2018a; Woodbury-Smith and Scherer 2018; Quesnel-Vallières et al. 2019; Vicari et al. 2019). Our aim in this review is to consider how recent findings are shaping our understanding of autism and how discoveries might inform clinicians.

The concept of autism has gradually broadened since the time of Leo Kanner’s first clinical descriptions in his 1943 seminal paper (Harris 2018). The prevalence of autism remained low for very many years but has risen over the last few decades from around 2–4 in 10,000 to an estimate of 1%. This is thought to reflect changes in ascertainment and the broadening of diagnostic criteria (Rutter 2007; Rutter 2011, 2013a); these issues are important to consider when we come to interpreting genetic study findings. Both DSM-5 (APA 2013) and ICD-11 (WHO 2019) now use the umbrella term “autism spectrum disorder”. Another consideration is how we deal with monogenic disorders. Previously Rett syndrome (RTT) was considered as a form of autism that affects females. However, there are some key clinical differences from typical autism, in that it is a progressive neurological disorder with very characteristic features including loss of purposeful hand use and repetitive movements. Rett syndrome is now known to be caused by variants in the methyl-CpG binding protein 2 (MECP2) gene. Given its distinctive clinical presentation and single known cause, RTT appropriately is no longer grouped with autism in DSM-5 and ICD-11. There are an additional group of monogenic disorders, such as Tuberous Sclerosis and Fragile X syndrome, that have very distinctive physical features (e.g. tubers) and which can be accompanied by autism. Readers interested in the clinical features of these disorders and research on monogenic disorders that has moved from gene identification to reversal of deficits in animal models are directed elsewhere (Sztainberg and Zoghbi 2016).

Some consider these disorders as syndromal autism or high penetrance forms of autism. As we will discuss later however, new genetic and biological findings have highlighted that there is no clear-cut distinction between rare monogenic and common multifactorial autism.

## The Heritability of Autism: From Early to Modern Twin and Family Studies

Although Kanner is reported to have viewed autism as an innate disorder (Rutter 2013; Harris 2018), a strong psycho-analytic tradition led to the growing belief that “refrigerator” mothers might be to blame. The first twin study of autism conducted by Folstein and Rutter (Folstein and Rutter 1977) was ground-breaking because it clearly showed a predominantly genetic contribution to autism. The most recent meta-analysis of all published twin studies of autism/autism spectrum disorder conducted by Tick and colleagues (Tick et al. 2016) also yielded a large heritability estimate of 64–91% and no significant shared environmental contribution. These authors demonstrated that if the estimated prevalence rate of autism is incorrectly specified for the study population (1% instead of 5% which is the appropriate figure for a broader autism phenotype), this essentially results in an increased non identical (dizygotic DZ) twin correlation but does not affect identical (monozygotic MZ) twin correlations, thereby resulting in a reduced heritability estimate and a stronger shared environmental contribution. Thus the shared environmental contribution observed in two outlying studies (Hallmayer et al. 2011; Frazier et al. 2014) appeared to be explained by the assumption of prevalence and an overinclusion of concordant DZ twins. The study by Tick and colleagues is also important in showing that if the autism broad phenotype is clinically recognised, then that ought to be taken into account by assessing different thresholds when fitting statistical models.

A more recent study combined extensive family and twin population-based data across five different countries: Denmark, Finland, Sweden, Israel and Western Australia (Bai et al. 2019). The authors again observed a high median heritability of 80.8% for autism with only modest country-specific variation in estimates varying from 50.9% in Finland to 86.8% in Israel. Shared environment contributions were negligible. The authors conducted sensitivity analyses on Finland and Western Australia because these yielded lower heritability estimates when compared to the other countries. They further showed that a random under-ascertainment of autism may result in an underestimate of true heritability and increase the observed shared environment contribution. This study by Bai et al. also examined maternal contributions to autism that was enabled by the inclusion of offspring from sisters. Surprisingly perhaps, given the hypothesised role of prenatal exposures and risk for autism, there was negligible maternal contribution to autism risk. This observation replicated a previous Swedish study that had also observed limited maternal contributions to autism (Yip et al. 2018). However, the authors acknowledge, that the design could only examine genetic factors shared by sisters and other types of design are required to robustly assess the contribution of early life exposures. Taken together, all these twin studies provide strong evidence of a mainly genetic contribution to autism and negligible shared environmental effects.

## What Family and Twin Studies Have Told us About the Autism Phenotype

Twin and family studies of autism were important in showing early on that the biological relatives of probands with autism were not just at heightened risk for autism itself but also showed elevated rates of milder autistic-like features. This led to the appreciation of there being a broader autism phenotype characterised by features like those of autism but less severe than in affected individuals (Le Couteur et al. 1996).

Family studies have suggested that familial liability to autism explains the higher rate of pragmatic language difficulties (Miller et al. 2015), social abnormalities and unusual personality features such as shyness and aloofness in relatives of probands with autism (Le Couteur et al. 1996). However, the broader phenotype is different to autism in several key aspects. First, it is not associated with epilepsy, second there is no association with lower IQ or specific learning problems. Although now there are ways of assessing the broader autism phenotype (de Jonge et al. 2015), a key challenge lies in knowing where its boundaries lie given that autism genetic liability appears to operate across a continuum and confers risk for a range of other neurodevelopmental and psychiatric disorders which we will discuss later.

Another striking finding from twin studies was the observation of very high variability in the clinical features of autism (e.g. IQ, clinical symptoms) among MZ twins who share all their inherited DNA (Le Couteur et al. 1996). This suggests that the clinical manifestation of autism even given the same level of genetic liability maybe subject to stochastic factors or environmental factors that are not shared by MZ twins. It has been argued that we should not be surprised by chance or stochastic events as contributors to health and disease, given they are likely to have important evolutionary advantage (Davey Smith 2011).

The third clinically relevant finding that emerged from autism family studies deals with clinical indices of genetic heterogeneity. In general, more severe autism (indexed by autistic symptom severity or lower verbal IQ- a measure of overall language/communication skills) has been observed to be associated with greater familial loading (Rutter 2000). However, there was interest in whether familial loading was different for probands who also showed profound intellectual disability (global intellectual and adaptive functioning difficulties); that is, whether profound intellectual disability indexed a discontinuity in terms of genetic liability for autism. That is important in refining estimated recurrence risks of autism in affected families. Family study findings here have been mixed with the largest study suggesting that the discontinuity in terms of familial loading appears to apply to autism accompanied by very severe language deficits (Pickles et al. 2000).

A final issue that has been studied is whether autism should be viewed as a discrete diagnostic entity. Although for clinical purposes autism is defined categorically, it can also be viewed as a continuously distributed dimension. Twin studies have been used to investigate the validity of a dimensional approach by examining whether “disorder” lies at the extreme of a dimension. Several twin studies have utilised population data on autism to assess this. Most have suggested that heritability estimates are consistent across the typical population range and extreme autism scores or show a strong genetic correlation between autism trait and diagnosis (Lundström et al. 2012; Colvert et al. 2015). However, one study (Frazier et al. 2014) yielded different findings where autism showed higher heritability at the high end of the continuum when compared to low scorers. However, as already discussed, the twin sample here was highly selected rather than population-based. The most recent and largest population-based study examined scores on the Childhood Autism Spectrum Test (CAST) at age 8 years in 2,256 MZ twin pairs and 4157 DZ pairs (Tick et al. 2016). Here, heritability estimates for high autism scores were not substantially different to those with low scores. Another recent large twin study in Sweden also showed a modest genetic correlation (0.48 (95% CI 0.44–0.53),) between autism and a trait measure of autism (Taylor et al. 2019a, b). Thus, so far most of the twin research suggests that autism can be viewed as lying on a continuously distributed dimension in the population as well as a category for clinical purposes and that these are similarly heritable and share similar although not identical genetic contributions.

These findings together with family study observations of the broader autism phenotype highlight that there is no clear-cut boundary that demarcates a diagnosis of autism or autism (Rutter and Pickles 2016). Although rigorous diagnostic instruments such as the ADI and ADOS are invaluable for research, extremely lengthy protracted assessments in practice that search for an “accurate” diagnosis thus are not justifiable when intervention is a priority.

That is not to say, we do not value careful assessment, but rather that there needs to be an appreciation that dimensional and categorical approaches both are valid and that the diagnosis of autism cannot be defined accurately as a disease with discrete boundaries no matter how many assessments are conducted (Rutter 2011).

## Autism Overlap with Other Childhood Neurodevelopmental Disorders

It is now well-recognised that autism shows a high level of comorbidity and population -based twin studies have consistently observed that autism traits show strong genetic correlation with other neurodevelopmental traits and diagnoses (Thapar and Rutter 2015a). A twin study in Sweden for example, showed that autism was not only highly heritable but that three quarters of its genetic variance was shared with ADHD and that genetic factors also contributed to the overlap between autism and learning, motor co-ordination problems and tic disorders (Lichtenstein et al. 2010).

A subsequent analysis of Swedish Registry family data further highlighted important links between autism and ADHD (Ghirardi et al. 2017). This study included 899 654 individuals in Sweden with diagnoses recorded nationally by clinical services. The authors observed that those with autism were at higher risk of having ADHD compared with individuals who did not have autism (odds ratio (OR) = 22.33, 95% confidence interval (CI) 21.77–22.92). Almost half the individuals with autism also received a diagnosis of ADHD. They further established that the monozygotic co-twins of those with autism showed an increased risk of ADHD (OR = 17.77 95% CI 9.8–32.22) compared to dizygotic co-twins (OR = 4.33 95% CI 3.21–5.86). These associations were most prominent for those with higher functioning autism rather than low functioning autism (with intellectual disability). The findings highlight that while relatives of those with autism have long been known to be at elevated risk for autism and the broader autism phenotype, they also are at high risk for ADHD and other neurodevelopmental disorders. That is, autism genetic liability can manifest not just as autism but also as ADHD and other neurodevelopmental disorders. These overlaps will be further considered in the light of molecular genetic studies. Observations from family and twin studies however do lend weight to the stance taken by both DSM-5 and ICD-11 in grouping child neurodevelopmental disorders and in now enabling ADHD to be co-diagnosed with autism.

## Gene-Environment Interplay

Although autism is highly heritable, it is not entirely explained by genetics, environmental factors also contribute. The role of environment in autism risk has been reviewed extensively elsewhere (Mandy and Lai 2016). Here we will consider how environmental risks might work together with genetic liability.

It now is known that many environmental risks are correlated with genetic liabilities and thus, some of the prenatal and early life factors that have been observed to be associated with autism potentially could arise through gene-environment correlation (Rutter 2015). For example, passive gene-environment correlation would arise when a mother’s genetic background influences environmental exposures associated with autism risk, such as medical conditions or behaviours in pregnancy (e.g. dietary intake of folic acid). Maternal genetic liability for ADHD has been shown to be associated with many prenatal exposures; for example, smoking in pregnancy (Thapar et al. 2009; Thapar and Rice 2020). However, thus far, similar findings have not been observed for maternal autism genetic liability (Leppert et al. 2019). One issue that requires discussion relates to the observation that older maternal and paternal age or delayed paternity are associated with risk for autism and older paternal age also has been linked to a higher risk of spontaneous or de novo rare variants. Such variants have been observed to contribute to autism risk (see later). However, recent genetic epidemiological findings suggest that age-related de novo variants do not appear to be a primary explanatory mechanism for the paternal age findings (Gratten et al. 2016); and in one study it was estimated that shared genetic liability between father and offspring could contribute to the association (Gratten et al. 2016). This is an important issue for older fathers who are concerned about risk of autism in offspring. Interestingly, although autism shows such strong comorbidity and shared genetic liability with ADHD, it is younger rather than older parental age that is associated with ADHD.

Active and evocative gene-environment correlation arise when the offspring’s genetic liability is associated with an environmental exposure; for example, where an individual seeks out specific environments or evokes environmental exposures depending on their genetic propensity. Children with autism for example, are at higher risk for maltreatment and bullying victimisation (Hoover and Kaufman 2018; McDonnell et al. 2019). Genetic studies suggest that these exposures are correlated with background family and genetic liability (Dinkler et al. 2017; Ohlsson Gotby et al. 2018). These adversities could arise from both passive gene-environment correlation (e.g. via parental neurodevelopmental impairments) or evocative gene-environment correlation (child genetic background). The findings highlight that genetic and environmental influences are not independent of each other. For clinicians, the phenomenon of gene-environment correlation means that where social adversity accompanies autism, it does not necessarily mean that the social adversity was causal or that the autism is a different type of adversity-related autism. While early social adversities, unless unusually extreme (Rutter et al. 2007) have not been demonstrated to be causal for autism (Dinkler et al. 2017) -they do have important risk effects on depression and could provide one explanation for phenotype and genetic links observed between autism and depression (Thapar and Rutter 2019). However, that requires explicit investigation.

Gene-environment interaction is a different concept that refers to the phenomenon where the effect of environmental exposures on phenotype is modified by the background genotype or genetic liability. Although shown to contribute in animal studies (Thapar and Rutter 2015b, 2019), to date convincing findings that gene-environment interaction contributes to autism risk have been lacking.

## Molecular Genetic Approaches to Understanding Autism

The last decade has witnessed an enormous surge in published molecular genetic findings on autism. Genome-wide studies across medicine, psychiatry and the social sciences have involved the interrogation of genomic variation to search for links between specific variants and disorder or traits. Genomic variation can be characterised by its population frequency as well as by whether the variation involves DNA structure or sequence (State and Thapar 2015). Genome-wide association studies (GWAS) involve comparing the frequencies of hundreds of thousands of common gene variants, known as single nucleotide polymorphisms (SNPS; frequency > 5%) in cases and controls (Sullivan et al. 2018). Given the very large number of statistical tests required for so many variants and because common variants each have small effect size (e.g. odds ratio 1.1–1.2), extremely large sample sizes have been required to detect genome-wide significant variants. Other genome-wide studies have examined the contribution of rare structural and sequence variants that have larger effect size using family-based designs as well as case–control cohorts. Rare DNA variants are sometimes referred to as mutations in the literature although there are recommendations the term variant should be used (Richards et al. 2015).

Autism genetic liability can be viewed as a risk continuum in the population where those with clinical disorder lie at one extreme of this liability curve. Common gene variants appear to contribute to most of the population risk; environmental and stochastic influences will also contribute and as we will discuss, rare variants act against a background of these other influences to shift individual liability along the risk continuum towards disorder.

## Common Gene Variant Contribution to Autism

Although autism is highly heritable, and despite common gene variants having been considered to contribute substantially to population risk, individual variants have only recently been identified. The most recent genome-wide meta-analysis of 18,381 people with autism and 27, 969 controls led to the identification of five genome-wide significant loci (Grove et al. 2019). The problem with GWAS is that genome-wide significant findings represent just the start because it does not identify causal genes or mechanisms; much further work is needed to uncover what genes are likely causal and how gene variation leads to disorder. Also, SNPs only capture a very small proportion of the total genetic variance as a result of which SNP heritability for autism is low (0.118) and common genetic liability has no predictive utility at present. However, GWAS findings do highlight that common as well as rare variants contribute to the genetic architecture of autism. Also, there is growing interest in using GWAS to generate composite measures of common gene risk variants nominally associated with a given disorder, known as polygenic risk scores. In other areas of medicine, polygenic risk scores when combined with clinical variables are being considered as potentially useful predictors of disease onset, for example in high-risk groups, and for estimating prognosis (Lewis and Vassos 2020). Thus, it is plausible that with larger GWAS discovery samples sizes and more powerful PRS, these could when combined with other measures, have clinical utility in the future.

The authors of the largest autism GWAS further interrogated the Danish registry ICD-10 diagnostic data for the Danish iPsych cohort that included 13,076 cases and 22,664 controls. They observed SNP heritability was three times higher for autism without intellectual disability than for those with autism who also had intellectual disability. These findings are difficult to equate to those from twin study findings because twin heritability includes all inherited genetic variation although some family studies had suggested higher familial loading in less intellectually or language impaired probands. Intriguingly there was also some suggestion of possible heterogeneity across the different ICD-10 diagnostic subgroups (e.g. Asperger’s, atypical autism) but caution is required about these findings because they have not been replicated and are based on clinically ascertained subjects.

One of the most striking findings about autism common genetic liability is that it shows a strong positive genetic correlation with IQ and educational attainment. This is puzzling given that autism itself is associated with lower IQ. The observation is not explained by the artefact of selection bias or population stratification effects because when parent offspring trios are examined, over-transmission of alleles associated with higher educational attainment is observed in affected vs. unaffected siblings (Weiner et al. 2017). These findings are a puzzle and this relationship with educational achievement is very different to the pattern observed for neuropsychiatric disorders. For example, ADHD and schizophrenia, as expected, show a negative genetic correlation with IQ and educational achievement. Prior to the advent of GWAS findings, it was well recognised that around a third of those with autism have been reported to manifest outstanding cognitive skills, so called “savants” (Howlin et al. 2009). Other striking clinical findings include the observation that, a proportion of those with autism show early language regression and that associated epilepsy typically onsets in adolescence (Rutter and Pickles 2016). How these clinical observations link to the recent genetic findings on autism and IQ remains unknown.

What has emerged consistently from GWAS of autism and psychiatric disorders is evidence that genetic influences transcend diagnostic boundaries, in keeping with findings from twin and family studies. The most recent meta-analysis of eight psychiatric/neurodevelopmental disorders that included anorexia nervosa (AN), ADHD, autism, major depression, obsessive compulsive disorder (OCD), schizophrenia (SCZ) and Tourette syndrome (TS) observed substantial pleiotropy with over 100 loci associated with more than one disorder and prominently involved in neurodevelopment and expressed in fetal life (Lee et al. 2019). Interestingly autism showed strongest genetic correlations with ADHD (rg = 0.44), depression (rg = 0.45) and to a lesser extent with schizophrenia (rg = 0.22). Yet autism unlike any of these disorders is not amenable to treatment by medication and even those that do show improvements do not respond to the same treatments (e.g. stimulants for ADHD, SSRIs for depression and atypical antipsychotics for schizophrenia). Some of the pleiotropic loci, including two shared between SCZ and autism showed evidence of opposite direction effects and autism was implicated in 36% of the pleiotropic loci.

Interestingly the authors utilised the genetic observations to investigate the structure of different psychiatric disorders using exploratory factor analysis. This identified three correlated factors: one comprised disorders characterised by compulsive/perfectionistic behaviour (AN, OCD and more weakly TS), the second factor included mood disorders and psychosis (depression, bipolar, schizophrenia) and the third factor encompassed neurodevelopmental disorders (autism, ADHD and TS) but surprisingly also depression (Lee et al. 2019). This structure is interesting because it does argue in support for the DSM-5 grouping of placing autism and ADHD together under neurodevelopmental disorders. However, the prominent genetic overlaps fuel the argument that diagnostic classification ought not be reified.

Another finding from GWAS is that autism diagnosis genetic liability as captured by common variants (using linkage disequilibrium (LD) score regression and polygenic risk scores) shows overlap with population social-communication traits (Robinson et al. 2016; St Pourcain et al. 2018) and autism traits (Taylor et al. 2019a, b). Thus, molecular genetic studies converge with twin study findings in suggesting that autism lies at the extreme of a continuum.

## Rare Genetic Variants

In contrast to GWAS findings, autism investigations of rare genetic variation (< 1% frequency) have yielded many more discoveries so far. Generally rare variants tend to show larger effect sizes relative to common variation. Initial whole genome rare variant searches focused on a type of variation known as copy number variation (CNVs). These copy number variants are regions of DNA containing thousands to millions of base pair variants (the building blocks of DNA) that are duplicated or deleted relative to a reference genome. These deletions and duplications can span many different genes and although large they are too small to be seen by light microscopy. More recent sequencing studies have focused on rare variants that involve changes to a single base pair known as single nucleotide variants (SNVs) and insertion or deletion of base pairs (indels). Rare variants can be transmitted from parent to offspring (inherited) but also can be de novo in origin where the variant first arises in the parent germline (oocytye or spermatozoa) or later, after fertilization when they are known as post zygotic somatic variants (State and Thapar 2015; Lim et al. 2017). All these variants appear to contribute to autism risk.

### Copy Number Variants

Genome-wide searches for rare variants associated with autism risk have involved simplex families where only one proband is affected, consanguinous as well as multiplex families where multiple siblings are affected. It is worth recognising that such designs that enhance variant discovery may mean that cases included are not necessarily typical of every clinician’s clinic group.

An initial study conducted by Sebat et al. 2007 (Sebat et al. 2007) involved 264 families, including 118 “simplex” families containing a single child with autism, 47 “multiplex” families with multiple affected siblings, and 99 control families with no diagnoses of autism. The authors identified an increased burden of rare de novo chromosomal structural variants consisting of deletions and duplications (copy number variants; CNVs) in individuals with autism when compared with healthy controls (1% rate); they observed a de novo CNV rate of 10% in simplex cases and 3% in cases from multiplex families. Subsequent studies observed similar findings with an increased rate of rare de novo CNVs in autism especially in simplex families (Marshall et al. 2008; Sanders et al. 2011).

What has emerged clearly from these studies is the high degree of etiological heterogeneity for autism even within families which is in keeping with family and twin study observations. The same variant does not necessarily manifest in two affected siblings with autism. Nevertheless there are some recognised recurrent autism associated de novo CNVs (Sanders et al. 2015). Replicated CNV regions include 1q21.1, 3q29, 7q11.23, 16p11.2, 15q11.2–13 and 22q11.2 (Sanders et al. 2015). Copy number variants typically encompass multiple genes so while de novo CNVs are thought to have a high probability of being causal, we cannot deduce the mechanisms that lead to autism without further investigation. Also autism-associated CNVs are highly pleiotropic, with many of the same CNVs also being associated with risk for intellectual disability, schizophrenia and ADHD (Williams et al. 2010; Marshall et al. 2017; Chawner et al. 2019).

### Sequencing Studies

Recent genetic investigations of autism have focused on sequencing all DNA variation within the coding region of the genome (exome). Exome sequencing studies of simplex families and case–control comparisons have observed de novo and inherited rare variants associated with autism risk. The study by Sanders et al. 2015 (Sanders et al. 2015) combined analysis of de novo CNVs, and variants identified from exome sequencing, that included indels (small insertions and deletions) and single nucleotide variants (SNVs), and yielded 71 autism risk loci. Findings from the largest autism exome sequencing study to date involved analysing 11,986 autism cases that included 6, 430 proband-parent trios and 5556 cases with 8809 controls. Integrating and analysing these data has led to the implication of 102 autism risk genes (Satterstrom et al. 2020). The authors observed a significant 3.5 fold increase in de novo protein truncating variants (PTVs) and a non-significant 1.2 fold enrichment of inherited PTVs.

With the advent of whole genome sequencing (Yuen et al. 2017; Werling et al. 2018), the number of implicated genes is set to rise further to several hundreds at least (Sestan and State 2018). Initial findings suggest possible contributions from non-coding variants as well as tandem repeat sequences (Trost et al. 2020) (repeated sequences of nucleotides such as seen in Fragile X syndrome). However as whole genome sequencing involves interrogating many more variants than whole exome sequencing, even larger sample sizes will be required to yield high confidence genetic discoveries (Searles Quick et al. 2020).

There are several observations that emerge from these rare variant studies of autism. First, for autism, there has been a much greater discovery rate of rare variants compared with common variants. The under-identification of autism common gene variants likely is due to much smaller sample size availability than for other disorders (e.g. schizophrenia, hypertension) because investigations suggest that at a population level, polygenic inheritance remains an important contributor to population risk for autism. Moreover polygenic variation still appears to contribute additively to risk of autism in those who possess a strong de novo variant (Weiner et al. 2017). However, in clinics where the cohort includes affected individuals only rather than the whole population, there is enrichment for rare variants. It is estimated that around 10–40% of individuals diagnosed with autism could be explained by de novo rare variants (Sestan and State 2018). However, de novo variants cannot explain the familial and genetic aggregation of autism.

The second issue is that unlike common variants, the detected rare variants have larger effect size (e.g. odds ratio of > 20 (De Rubeis et al. 2014)) although penetrance for many mutations appears to be highly variable. Some have proposed that individuals with the same variant may show clinical heterogeneity because of “second” or “multiple” hits where additional variants modify the clinical picture by adding to risk or having a protective effect.

This makes it difficult to predict risk for the purpose of genetic counselling (see later). Large effect size and deleterious de novo variants (e.g. result in loss of function of the gene product) are over-represented in those with autism yet would be subject to natural selection where they tend to be removed from the gene pool over generations. This would explain why in general a higher rate of these variants has been observed in simplex families.

A third observation is that autism associated de novo rare variants although over-represented in those with comorbid intellectual disability are present across the spectrum of intellectual ability. This means that rare variants are still relevant for higher IQ individuals with autism but this group may not be a high priority for genetic testing (see genetic testing later).

A fourth point relates to the male preponderance in autism. Family and twin studies originally suggested that the siblings of females with autism are at higher risk for autism than the siblings of males (Robinson et al. 2013). This suggested that females might in some way be protected against developing autism despite inherited liability. Investigation of rare variants are also consistent with the female protective effect hypothesis as a mechanism for the increased male prevalence of autism because affected females have been observed to also carry an increased burden of de novo variants.

Finally, what is very clear from molecular genetic studies is that autism is not only clinically heterogenous, it is highly heterogeneous in terms of genetic etiology at a molecular level and autism-associated rare variants like common variants are pleiotropic. Autism-associated de novo CNVs are associated also with schizophrenia risk (e.g. 22q11 microdeletion), intellectual disability and ADHD (e.g. 15q 11.13) (Chawner et al. 2019). Furthermore, initial whole genome sequencing suggests that in multiplex families, more than half of the affected siblings carry different autism related variants (Yuen et al. 2015). Nevertheless, as we will discuss, most scientists who work in this area are optimistic that clinical translation is feasible (Sestan and State 2018; Quesnel-Vallières et al. 2019; Wiśniowiecka-Kowalnik and Nowakowska 2019). However, we view that a lack of detailed clinical information beyond simply autism diagnostic interviews may be one key barrier in translating genetic findings into clinical practice. Detailed clinical descriptions as well as physical investigations and imaging data will help us better understand and characterise the different variants and enable clinicians to interpret their clinical and long-term impacts and such studies are underway (D’Angelo et al. 2016).

As rare variants, especially de novo ones are often idiosyncratic to families, and multiple different common and rare variants contribute risk, we do not know as yet whether there are common final common developmental and biological pathways to autism that could be ultimately targeted safely by treatment at the appropriate developmental stage. We will discuss this next.

## From Genes to Biology and Treatment

A strong motivation for identifying autism risk genes is to provide insights into its at present unknown biological underpinnings, pathogenesis and to pave the way for treatment. Rare variants are considered attractive for potentially providing clues into potential biology because of their large effect size and especially de novo variants that appear causal. However, the problem is that rare variants do not act in isolation in any given affected individual (e.g. polygenic background), there are so many genes involved and variants are pleiotropic. Also, so far, common variants and rare variants have not been definitively shown to converge on the same biological systems and we do not know whether different rare variant carriers show a similar type of autism and underlying biology to each other and to those who do not carry a rare autism variant at all.

There is growing interest however in examining how different gene variants converge on the same gene expression and protein networks to identify potential key biological pathways that underlie autism. These approaches have also involved examining how gene variants impact on different brain cell types, in different places across the brain and at different developmental periods. A growing number of bioinformatic resources enable researchers to infer what identified gene variants do which is less costly and time intensive than examining the function of one gene variant at a time in model organisms and cellular models. Clearly with so many autism risk genes involved, identifying autism biological underpinnings is going to be complex especially as the phenotype manifestations of autism are not easily recapitulated by animal and cellular models. Nevertheless, experts in this area are optimistic that systems biological approaches that examine the convergence of autism associated genes, proteins, cells, circuit and behaviour will yield important biological insights. So far the autism associated risk genes implicate synaptic proteins, and those involved in chromatin and transcriptional (the conversion of DNA to RNA) regulation (Sestan and State 2018), are mainly expressed early in brain development during prenatal life and encode a very wide variety of proteins (Ruzzo et al. 2019); (Sestan and State 2018).

## Genetic Testing and Counselling

Research advances have led to a widespread appreciation now that genetic contributions are important in the etiology of autism as a result of which genetic testing and counselling have become salient to clinicians and affected families (see (Griesi-Oliveira and Sertié 2017); (Nurnberger et al. 2019)). Traditionally, where families have wanted to make reproductive decisions or were concerned about risk in siblings, the clinician has relied on recurrence risks reported in family studies. One challenge here is the reported estimates vary widely across studies and country (Jokiranta-Olkoniemi et al. 2016) and very much depend on the sample ascertained (e.g. whether simplex or multiplex families). Typically the rate of autism in siblings of probands has varied from between 10–15% (Vorstman, et al. 2017). However the risk of autism in siblings is higher if the proband is female, in keeping with the female protective effect, and is higher in male than in female siblings (Werling and Geschwind 2015; Jokiranta-Olkoniemi et al. 2016; Palmer et al. 2017). Also, the recurrence risk is much higher if two siblings already are affected, reported to rise to around 30–50% (Ozonoff et al. 2011; Werling and Geschwind 2015).

The other problem with recurrence risks is that the risk estimate is not individually tailored. This leads us to the question of molecular genetic testing. At present, there is no clinical rationale for testing common gene variants because of their limited predictive utility. However, the situation is different for rare variants. Cytogenetic testing and screening for syndromes such as Fragile X syndrome and Tuberous Sclerosis have long been part of routine clinical investigations when these syndromes are suspected. Chromosomal microarray investigations are now widely available as the first line of genetic testing across many countries.

Given the growing number of rare variants implicated in autism risk there are some potential benefits of further molecular testing. These include for example, more individually tailored recurrence risk estimates of autism, access to support groups, a greater understanding of how autism has arisen in the affected proband and enhanced early recognition and treatment of medical conditions known to be associated with the variant (e.g. occult congenital heart disease) as well as increased vigilance about comorbid psychiatric disorders (e.g. elevated risk of psychosis in those with a 22q11 deletion). In some countries e.g. the United States, guidelines currently recommend that all those with a diagnosis of autism are screened for CNVs using chromosomal microarrays (Schaefer and Mendelsohn 2013). In other countries including the UK, current guidelines (e.g. (“Overview Autism spectrum disorder in under 19 s: recognition, referral and diagnosis Guidance NICE” n.d.) do not recommend routine genetic testing for autism unless there is accompanying intellectual disability or dysmorphic features. At the same time, there is growing interest in the clinical utility of whole genome sequencing to identify deleterious rare variants (e.g. in unwell new-born infants) and health providers for some nations, including NHS England (not across all devolved UK nations), have expressed the intention of making whole genome sequencing a routine part of medical care.

However, there are certainly many challenges to genetic testing for autism. First, it is difficult to clinically interpret findings. Rare variants associated with autism risk show variable penetrance, expressivity and are highly pleiotropic (Rosenfeld et al. 2013; Kirov et al. 2014; Kirov 2015; Fernandez and Scherer 2017; Woodbury-Smith et al. 2017). This means that carriers of a given variant and relatives of those affected could remain healthy, show the same phenotype but with a very different level of severity or display a different phenotype altogether (e.g. ADHD or schizophrenia rather than autism), as we have already discussed.

There are studies which are investigating the effects of specific recurrent variants (e.g. 16p11.2) (D’Angelo et al. 2016). However, even for these, polygenic background and stochastic factors remain relevant influences on the phenotype. Also, inherited CNVs and SNVs may be presumed to have different implications for reproductive decisions than de novo variants. A further consideration for genetic counselling is whether de novo variant arises in the germline or after fertilization (somatic). Finally, as already mentioned, different autism associated de novo variants can occur in the same family (Yuen et al. 2015). Thus, providing accurate information to families is challenging.

Second, observed variants may be known to be associated with autism risk but it is not always clearly known if they are causal for a given individual. Third, it is important to consider potential negative aspects of genetic testing. For example, what are the impacts of a “negative” test where the clinician fails to detect a known pathogenic variant? Will that serve as a disappointment to expectant families who seek an answer for why their child has autism? Alternatively, for those who carry a rare variant or where one is inherited, will this have negative impacts that include guilt, shame, anxiety as well as potential detrimental effects on life insurance and life prospects including future health and reproduction? It has been highlighted that despite very rapid advances in genetic discoveries for complex disorders, this has not been accompanied by high quality clinical research on genetic testing in child health and psychiatry including how clinicians should be trained about this, how findings should be shared with families and what the clinical utility and long term risks and benefits of testing are. Overall, our view is that referral to clinical genetics services for investigation and counselling is appropriate for autism accompanied by ID or complex presentations (comorbid dysmorphic features, a medical condition) but we believe that judgement on referral will change rapidly and may depend on the local context and clinic case-mix. Future criteria and national decisions about referral will depend on findings that emerge not just from high-tech genetic discoveries but also through research on the clinical utility of genetic testing as well as on available healthcare and social resources especially as at present there is currently no evidence of cost effectiveness for genetic tests for autism (Ziegler et al. 2017).

## Conclusion

Much progress has been made in our understanding of the genetics of autism in the last 40 years. We know now that it is one of the most heritable of disorders and that typically it is multi-factorial in origin. Both common and rare genetic variants contribute to risk and there is strong interest in utilising gene discoveries to gain insights into the underlying biology of autism. However, autism shows enormous clinical as well as genetic heterogeneity. While the genetic discoveries represent a huge advance, there is a need to link this work with clinical research. Given the public interest in genetics, another pressing clinical issue is how genetic information is best shared with families and used in a way that is ethical and clinically useful.
